# *Acinetobacter baumannii* Bloodstream Infections: A Nationwide Study in Israel

**DOI:** 10.3390/microorganisms11092178

**Published:** 2023-08-29

**Authors:** Amir Nutman, Elizabeth Temkin, Liat Wullfhart, Vered Schechner, Mitchell J. Schwaber, Yehuda Carmeli

**Affiliations:** 1 National Institute for Antibiotic Resistance and Infection Control, Ministry of Health, Tel Aviv 6423906, Israel; 2 Faculty of Medicine, Tel Aviv University, Tel Aviv 6997801, Israel

**Keywords:** *Acinetobacter baumannii*, bloodstream infection, incidence, mortality, antimicrobial resistance, carbapenem resistance, population-based study

## Abstract

*Acinetobacter baumannii* (Ab) bloodstream infections (BSIs) are a major public health concern and associated with high mortality. We describe the nationwide incidence, antimicrobial resistance, and mortality of Ab-BSI in Israel using laboratory-based BSI surveillance data from January 2018 to December 2019. During the study period, there were 971 Ab-BSI events (508 in 2018 and 463 in 2019), with an average annual incidence of 8.08/100,000 population. The median age of patients was 72 (IQR 62–83), and 56.4% were males. Two-thirds of Ab-BSI events were hospital-onset (HO), with median day of onset 16 (IQR 9–30). HO-BSI incidence was 0.62/10,000 patient-days (rate per 10,000 patient-days: 2.78, 1.17, and 0.2 for intensive care, medical, and surgical wards, respectively). Carbapenem susceptibility was 23.4%; 41.4% and 14.9% in community and HO events, respectively. The 14-day, 30-day, and 1-year mortality were 51.2%, 59.3%, and 81.4%, respectively. Carbapenem-resistant Ab-BSI were associated with a significantly higher 14-day, 30-day, and 1-year mortality (*p* < 0.001 for all). In the multivariable model, age (aHR 1.02) and carbapenem resistance (aHR 3.21) were independent predictors of 30-day mortality. In conclusion, Ab-BSIs pose a significant burden with high mortality, especially associated with antimicrobial resistance. Attention should be focused on prevention and improving treatment.

## 1. Introduction

*Acinetobacter baumannii* (Ab) is a non-fermenting strictly aerobic, Gram-negative coccobacillus that has emerged from an organism of uncertain pathogenicity to an infectious agent of concern globally [1]. Ab has a remarkable ability to upregulate innate antibiotic resistance mechanisms and acquire new mechanisms through mobile-genetic elements [1,2], and isolates resistant to all available therapeutic options have been described with varying frequency [3,4]. In addition, due to its capacity to thrive under limited nutrient conditions, withstand desiccation, and develop resistance to disinfectants, Ab can endure for extended durations within the hospital environment. This endurance can lead to the emergence of hospital-based outbreaks that are challenging to eliminate and manage effectively [1,5,6,7]. In a global analysis of the burden of antimicrobial resistance in 2019, Ab was the fourth leading cause of deaths attributable to antimicrobial resistance [8].

The epidemiology of Ab infections include health care-associated pneumonia, bloodstream infections (BSIs), urinary tract infections (UTIs), and surgical site infections (SSIs) [1,9], particularly among debilitated patients in the intensive care unit (ICU) and residents of long-term care facilities that require ventilator care [10,11]. Risk factors for Ab infections include mechanical ventilation, intravascular devices, invasive procedures, prolonged hospital stay, admission to the ICU, and the use of broad-spectrum antibiotics, in particular third-generation cephalosporins and carbapenems [1,2].

Ab infections have also been described in the community, mainly in tropical climates, and among victims of natural disasters and war [1,12]. Although members of the *Acinetobacter* genus are ubiquitous in nature, environmental reservoirs of Ab outside the hospital have not been thoroughly investigated. Ab has been isolated from soil and vegetables collected from various settings, and from aquatic environments, animal healthcare settings, and human lice [12]. Ab isolates from white stork nestlings have been linked to human clinical isolates, suggesting an avian reservoir [13].

Risk factors for community-acquired Ab infections include living in tropical or subtropical climates, male sex, and underlying conditions including excessive alcohol consumption, smoking, diabetes, and chronic lung disease [14]. It has been suggested that excess alcohol consumption may promote the growth and virulence of Ab [14]. Community strains are generally more susceptible to antibiotics than hospital strains. The most common presentation was severe pneumonia, with other types of infections reported including soft tissue infections, endocarditis, meningitis, and urinary tract infections [14].

Ab is a frequent cause of nosocomial BSI. The Surveillance and Control of Pathogens of Epidemiologic Importance (SCOPE) Project in the early 2000s reported an Ab-BSI rate of 0.6 per 10,000 hospital admissions, making Ab the tenth most common cause of nosocomial BSI (1.3% of all nosocomial BSI events and 1.6% in the ICU), with a crude in-hospital case fatality rate of 34% [15]. In the SENTRY Antimicrobial Surveillance Program, Ab was the ninth most common pathogen causing hospital-onset (HO) BSI, responsible for 3.2% of HO-BSI between 1997 and 2016 [16]. In a previous nationwide study in Israel, Ab was the sixth most common pathogen causing HO-BSI, with an incidence rate of 8.4 per 10,000 admissions, and had the highest 30-day mortality (64.6%) [17].

In Israel, the National Institute for Antibiotic Resistance and Infection Control has been monitoring cases of BSI caused by sentinel bacteria across all acute care hospitals since 2012 [17,18]. This study’s objective was to outline the epidemiological patterns, offer a nationwide assessment of *Acinetobacter baumannii* bloodstream infections (Ab-BSIs), examine mortality rates, and evaluate the influence of antimicrobial resistance.

## 2. Materials and Methods

### 2.1. Study Participants

We analyzed data from laboratory-based surveillance covering the period between 1 January 2018 and 31 December 2019. These data were collected from all 29 acute care hospitals in Israel. The majority of blood cultures, regardless of where the BSI originated, are handled by laboratories within these acute care hospitals.

For this study, we considered all Ab-BSI events that were reported for adult patients aged over 18 years. We excluded one children’s hospital as well as a small community hospital that lacked adult internal medicine wards or intensive care units from our analysis.

### 2.2. Data Collection

The hospital laboratories provided monthly reports encompassing all blood cultures positive for Ab. These reports included patients’ unique identity numbers (UIDs), sex, date of birth, admission date, blood culture collection date, the specific ward where the culture was taken, and the results of antibiotic susceptibility testing (AST).

By utilizing patients’ UID, we conducted searches within the Ministry of the Interior’s death registry to retrieve dates of death. These data from the death registry were accessible up until 30 April 2020. Additionally, we obtained information about hospital admissions and the number of patient-days from the Israel Ministry of Health [19]. Population statistics were available from the Israeli Central Bureau of Statistics [20].

### 2.3. Laboratory Methods

Blood cultures underwent processing at the microbiological laboratories of the participating hospitals. These laboratories followed their respective routine procedures for identifying the isolates and conducting AST. As we only possessed laboratory reports, we lacked specific details about the identification systems utilized. Nevertheless, during the study timeframe (2018–2019), the Vitek^®^ 2 system (bioMérieux, Inc., Marcy-l’Étoile, France) was frequently employed for this purpose. Because this method cannot distinguish between *A. baumannii*, *A. nosocomialis*, and *A. pittii*, the three clinically relevant *A. calcoaceticus-A. baumannii* complex species [1], for the purpose of this analysis, we grouped these species together and refer to Ab in the broader sense of the Ab group.

### 2.4. Definitions

An Ab-BSI event was defined when Ab growth was detected in at least one blood culture. The sampling date of the first positive blood culture marked the onset of the Ab-BSI event.

BSI was categorized as either community-onset (CO) if the positive blood culture was obtained within the initial 3 days of hospitalization, or hospital-onset (HO) if the onset date was on the fourth day of hospitalization or later. This categorization followed the guidelines of the CDC (Centers for Disease Control and Prevention) for LabID events [21].

If there was another blood culture positive for Ab within 14 days of the initial onset, it was regarded as the same event. However, if this occurred after 14 days, it was considered a new event [21]. If a blood culture was positive with another microorganism within five days of the Ab-BSI, it was labeled as a polymicrobial BSI event. In the context of HO-BSI, the department where the Ab-BSI originated was determined based on the patient’s ward during the time the culture was obtained.

Antibiotic susceptibility was defined according to the first Ab isolate that marked the onset of the Ab-BSI event. This approach was in line with the method employed by EARS-Net (European Antimicrobial Resistance Surveillance Network) [22]. Ab isolates were classified as carbapenem-resistant (CRAB) if non-susceptible to imipenem or meropenem, and carbapenem-susceptible (CSAB) if susceptible to both. Susceptibility to other antibiotic groups was according to the international consensus definitions published by Magiorakos et al. [23]. Accordingly, Ab isolates were classified as multidrug-resistant (MDR) if non-susceptible to ≥1 agent in ≥3 antibiotic groups, extensively drug-resistant (XDR) if non-susceptible to ≥1 agent in all but ≤2 antibiotic groups, and pandrug-resistant (PDR) if non-susceptible to all antibiotic groups tested [23]. Polmyxin susceptibility was not included in the analysis due to technical issues with testing methods and interpretation of results [24].

### 2.5. Incidence

The incidence rates were calculated by determining the number of Ab-BSI events per 100,000 individuals in the population. For HO-BSI, the incidence rates were calculated as the number of events per 10,000 patient-days.

### 2.6. Mortality

We calculated mortality rates at different time intervals: 14 days, 30 days, and 1 year following the onset of the Ab-BSI. To perform these calculations, patients with missing or incorrect UID were excluded, as they could not be cross-referenced with the death registry.

For calculating the 1-year mortality rate, we considered only those patients who had a follow-up period of at least 1 year leading up to the date of our access to the death registry, which was 30 April 2020. In other words, we included patients with a BSI onset before 30 April 2019 to ascertain at least a full year of observation.

### 2.7. Statistical Analysis

Patient and Ab-BSI event characteristics were summarized as median and interquartile range (IQR) or percentage, as appropriate. The Wilcoxon rank-sum test was used to compare continuous variables, and chi-squared or Fisher’s exact test were used to compare categorical variables. We calculated incidence rates, 14-day, 30-day, and 1-year mortality with 95% confidence intervals (CI). To compare incidence between groups, we calculated relative risk (RR) with 95% CI. To compare 30-day mortality between HO and CO events and between CRAB and CSAB events, we used Kaplan–Meier survival analysis and a log-rank test. In order to assess the effect of carbapenem resistance on 30-day mortality, we used Cox regression analysis. Variables associated with 30-day mortality in univariate analysis (*p* < 0.1) were included in the multivariable model. Analyses were performed using SAS version 9.4 (SAS Inst., Cary, NC, USA) and/or SPSS version 29 (IBM Corp., Armonk, NY, USA).

### 2.8. Ethical Considerations

The study received approval from the jurisdictional institutional review board (IRB). The need for obtaining informed consent from participants was waived due to the analysis involving routinely gathered surveillance data.

## 3. Results

During the two-year study period, there were 971 Ab-BSI events, 508 events in 2018 and 463 events in 2019. Overall Ab-BSI incidence per 100,000 population was 8.08 (95% CI 7.57–8.59). Table 1 shows the incidence by year, sex, and place of onset. Overall incidence was higher in males, but the difference was non-significant for CO- BSI events. HO-BSI incidence was highest in ICU wards, and lowest in surgical wards.

Patient and BSI event characteristics for HO and CO-BSI events are presented in Table 2. Median age was 72 years (IQR 62–83), and 56.4% were male for all Ab-BSI events. Age was lower for HO vs. CO-BSI events (71 vs. 75 years, *p* = 0.01) and the percentage of males was higher (59.2% vs. 50.8%, *p* = 0.02). The majority of HO-BSIs were in medical wards (70%), and the median day of onset was 16 days (IQR 9–30).

Antibiotic susceptibility profiles for HO and CO isolates are presented in Table 2. HO isolates were significantly less susceptible to all antibiotics tested as compared to CO isolates. In HO events, isolates had an MDR phenotype in 88.5% of events vs. 65.4% in CO events (*p* < 0.001) and an XDR phenotype in 85.7% of events vs. 59.9% in CO events (*p* < 0.001).

A comparison of CRAB and CSAB-BSI events is presented in Table 3. The majority of CRAB-BSIs were HO (75.4% vs. 43.1% of CSAB, *p* < 0.001) and occurred in the large hospitals (>700 beds) group (68.6% vs. 53.6% of CSAB, *p* = 0.01). CRAB and CSAB displayed different seasonality patterns, with a higher percentage of CRAB-BSI during winter and spring (57% of CRAB vs. 33.8% of CSAB), and a higher percentage of CSAB during summer and autumn (43% of CRAB vs. 66.2% of CSAB, *p* < 0.001) Patients with CRAB were older (73 vs. 70 years, *p* = 0.01). Almost all CRAB isolates were XDR (99.2%) and 70.8% were resistant to all classes tested. Only 19.6% of CSAB were MDR, and only 6.2% were XDR.

**Figure 1:**
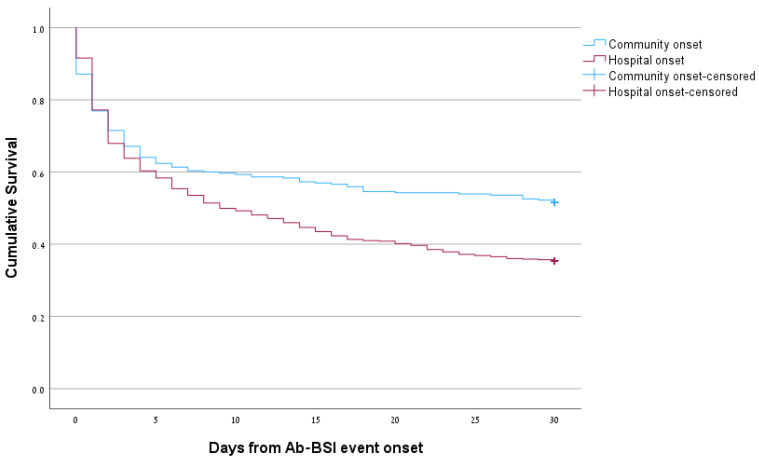
Kaplan–Meier 30-day survival curves for hospital-onset vs. community-onset *A. baumannii* (Ab) bloodstream infections (BSIs).

**Figure 2:**
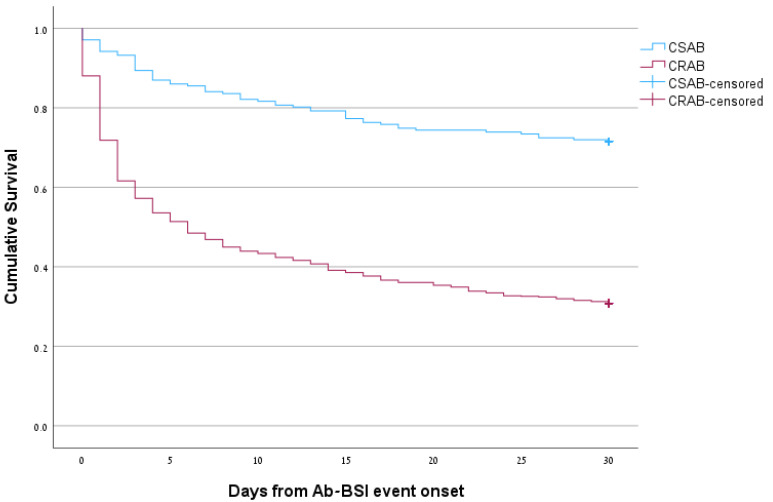
Kaplan–Meier 30-day survival curves for carbapenem-resistant *A. baumannii* (CRAB) vs. carbapenem-susceptible *A. baumannii* (CSAB) bloodstream infections (BSIs).

Data on mortality are presented in Table 2 (HO vs. CO) and Table 3 (CRAB vs. CSAB). Short-term mortality was extremely high and significantly higher for HO-BSI; 14-day mortality was 55.4% for HO-BSI vs. 42.7% for CO-BSI (*p* < 0.001) and 30-day mortality was 64.6% for HO-BSI vs. 48.5% for CO-BSI (*p* < 0.001). One-year mortality did not differ significantly between HO-BSI and CO-BSI (83.1% vs. 78.0%, *p* = 0.15). Mortality was higher for CRAB-BSI than for CSAB-BSI; 14-day mortality was 60.9% vs. 20.8% (*p* < 0.001), 30-day mortality was 69.2% vs. 28.5% (*p* < 0.001), and 1-year mortality was 88.6 vs. 55.4% (*p* < 0.001). Kaplan–Meier 30-day survival curves for HO-BSI vs. CO-BSI and CRAB-BSI vs. CSAB-BSI are presented in Figure 1 and Figure 2, respectively (*p* < 0.001 for both comparisons). In univariate analysis, age, place of onset, and ward type were significantly associated with 30-day mortality (Supplementary Table S1). Resistance to all antibiotic groups were significantly associated with 30-day mortality, however, for the multivariable model, only carbapenem resistance was included due to collinearity. In the multivariable Cox model, age (aHR 1.02, 95% CI 1.01–1.03) and carbapenem resistance (aHR 3.21, 95% CI 2.42–4.25) were independent predictors of 30-day mortality.

## 4. Discussion

This nationwide population-based study provided a national estimate of the burden of Ab-BSI in Israel for the years 2018–2019. The overall incidence of Ab-BSI was 8.08 per 100,000 population, and over two-thirds of cases were HO. Incidence of HO-BSI was lower in 2019 compared with 2018, while incidence of CO-BSI was slightly increased. Incidence was significantly higher in males for HO-BSI but not CO-BSI. HO events had higher antimicrobial resistance and higher 14-day and 30-day mortality than CO events. In the multivariable model predicting 30-day mortality, carbapenem resistance was significantly associated with 30-day mortality, while place of onset was not.

In a study published by Mun et al. [25] from two centers in Korea, Ab was the most frequent pathogen causing HO-BSI in patients who died within 2 weeks. In a previous study published by our group, Ab-BSI had the highest 14-day mortality among HO-BSI events, and was the second most frequent pathogen (after *K. pneumoniae*) in patients who died within 14 days [17].

In our study, incidence of HO-BSI was highest in the ICU, but the absolute number of events was highest in medical wards. Similarly, in the SENTRY study, most cases occurred in the internal medicine service [26]. To prevent the spread of Ab within hospitals, strict adherence to infection control practices, including hand hygiene, proper disinfection of equipment, and isolation precautions is essential. It is estimated that at least 50% of HO-BSIs are preventable with adequate infection control measures [27]. In a study by Rodríguez-Baño et al. [28], a prevention bundle that included screening, isolation of carriers, and increased attention to hand hygiene and environmental cleaning reduced MDR-Ab-BSI by 84%.

Data on CO-BSI from temperate climates are sparse. However, CO-BSI incidence is higher in tropical and sub-tropical regions. In a 1-year prevalence survey in Australia, 60.4% of Ab-BSI were CO [29]. In a population-based BSI surveillance study that included 20 hospitals in two rural provinces in eastern Thailand, *Acinetobacter* species bacteremia accounted for 3% of CO-BSI, with an overall incidence of 110 cases per 100,000 population [30]. Patients with community-acquired Ab-BSI had similar risk factors as patients with hospital-acquired Ab-BSI, but patients with CRAB-BSI were more likely to have medical devices (intravascular, urinary catheterization and mechanical ventilation) and previous use of antibiotics [31]. In another study of patients with CO *Acinetobacter* bacteremic pneumonia, 80% of patients required ICU admission; however, 28-day mortality was only 11%. Risk factors included smoking, alcohol abuse, chronic lung disease, diabetes, and chronic renal failure [32].

Carbapenems are considered the antimicrobials of choice for treating infections caused by susceptible Ab. CRAB are listed as an urgent threat by the CDC [33] and the WHO priority pathogen list [34]. In this study, carbapenem susceptibility was low: 14.9% in HO-BSI and 41.4% in CO-BSI. Ab susceptibility to carbapenems varies by geographic region. In the SENTRY study, meropenem susceptibility was 21% in the Asian Pacific, 22.2% in Europe, 13.7% in Latin America, and 54.9% in North America [16]. In a joint ECDC (European Centre for Disease Prevention and Control) and WHO report, carbapenem resistance varied widely in the European region, and was above 50% in 21 of 38 (55%) reporting countries (range: 0–96.4%); only 3 countries had resistance below 1%. In a population-weighted analysis, carbapenem resistance in Europe increased from 32.6% in 2016 to 38% in 2020 [35].

In this study, 75.4% of CRAB-BSI vs. 43.1% of CSAB-BSI were HO. CRAB-BSIs were associated with an over two-fold risk of death within 30 days: 69.2% vs. 28.5% for CSAB-BSI. In a prospective cohort study by Lee et al. [36], which included 10 hospitals in Korea, 85% of CRAB-BSI vs. 60% of CSAB-BSI were hospital acquired. Severe sepsis and inappropriate empirical therapy were more frequent for CRAB vs. CSAB BSI (53.2% vs. 21.6% and 75% vs. 19.3%, respectively), and all-cause 30-day mortality was higher (57.5% vs. 15.5%). Carbapenem resistance was independently associated with treatment failure in patients receiving inappropriate (aOR 6.17) or appropriate (aOR: 4.15) empiric antibiotics. In a study by Chusri et al. [31], 30-day mortality was similar for community-acquired vs. hospital-acquired CSAB (17% vs. 18%) but significantly higher for hospital-acquired CRAB-BSI (65%). Similarly, length of stay and total hospital costs were higher for hospital-acquired CRAB-BSI. Factors associated with 30-day mortality were infection with CRAB, inappropriate empirical antibiotic therapy, and higher Acute Physiology and Chronic Health Evaluation (APACHE) II score. However, in a single-center study from Greece, which included ICU-acquired Ab-BSI, mortality was similar between patients with CRAB-BSI and CSAB-BSI (43.3 vs. 46.9%) [37].

Because our study was based on laboratory surveillance, we did not have data on treatment. Previous studies have shown that appropriate empiric antibiotic treatment for Ab-BSI was associated with lower ICU mortality, with inappropriate treatment mainly driven by CRAB infections [38,39,40]. In a study by Shorr et al. [39] among ICU patients with Ab-BSI, CRAB was the strongest predictor of inappropriate empiric therapy, which was associated with in-hospital death. In a study by Park et al. [41], adequate antibiotic treatment within 3 days of BSI was a preventive factor for 14-day mortality only in patients who received non-colistin therapy. However, in a study by Al-Dorzi et al. [38], appropriate empirical treatment (mostly intravenous colistin) was associated with lower ICU mortality. In a study by Wenzler et al., shortening time to Ab identification along with a pharmacist-led antimicrobial stewardship intervention reduced time to effective therapy and improved clinical cure rates [42].

Seasonal variation in Ab infections has been described, with peaks during summer months [43,44]. In a study by Eber et al. [45], Gram-negative BSIs were more frequent during summer months, and *Acinetobacter* species showed the greatest seasonal variation, with a 51.8% increase in BSI frequency in summer months as compared with winter months. In a recent systematic review, a summer peak was consistently observed in different geographic regions and settings (CO and HO including ICU) for any Ab infection and Ab-BSI [46]. The hypothesized underlying mechanisms were increased colonization of humans (patients as well as healthcare workers) during warmer months, increased survival of Ab in environmental reservoirs, or that higher temperatures may increase the virulence of *Acinetobacter* species. Non-weather-related factors may include understaffing and lower adherence to infection control measures due to summer vacations [47]. In our study, the highest frequency of Ab-BSI was in the winter and autumn months, with no significant difference between HO-BSI and CO-BSI in seasonal variation. However, seasonal variation was related to antimicrobial resistance; the frequency of CRAB-BSI was highest during winter and spring (57% of all CRAB infections occurred during these seasons), while the frequency of CSAB-BSI was the highest during summer and autumn (66.2% of all CSAB infections occurred during these seasons).

Frequently, surveillance systems are limited to specific university or tertiary care hospitals that admit complex and severe cases, potentially leading to a bias in patient representation [48]. National surveillance systems offer an advantage by encompassing a diverse range of hospitals, including different types and locations, resulting in a more comprehensive view [49]. Our study’s strengths lie in its national scope and the utilization of a nationwide death registry, ensuring accurate monitoring of post-hospital discharge mortality. Our surveillance system covers all hospitals across Israel, spanning various bed capacities and academic affiliations. This inclusive approach ensures a wide representation of cases, minimizing potential bias, and thus provided extensive data on nationwide incidence, mortality, and antimicrobial resistance.

Our study has a number of limitations. Due to the absence of clinical data, the mortality figures provided herein encompass all causes of death rather than focusing solely on mortality linked to infections. Although survival data were not attainable for all patients, we believe that this omission would not significantly influence our mortality estimates. Additionally, we lacked information regarding prior hospitalizations either in acute-care or long-term-care settings. Consequently, we could not differentiate between healthcare-associated CO-BSI and cases of Ab-BSI that were genuinely acquired within the community. This in part may explain the high burden of antimicrobial resistance in CO events. Since some commercial identification systems employed in hospital microbiology laboratories cannot reliably differentiate between *A. baumannii* and other *A. baumannii* group species (*A. pittii* and *A. nosocomialis*), for the purpose of this analysis, these species were grouped together. However, this grouping is logical, as from a clinical and infection control point of view, these species exhibit similar behavior.

In summary, this comprehensive nationwide study underscores the significant prevalence of Ab-BSI within both hospital and community settings in Israel. It also sheds light on the notable link between antimicrobial resistance and mortality rates. These findings emphasize the critical need for targeted and concentrated infection control measures for the prevention and mitigation of Ab infections in general and BSI specifically, and also for enhancing the management of these highly impactful infections through improved treatment strategies.

## Figures and Tables

**Table 1:** Incidence of *Acinetobacter baumannii* bloodstream infections, by year, sex, and place of onset.

	Study Period (2018–2019) IR (95% CI) N = 971	RR (95% CI)	2018 IR (95% CI) N = 508	RR (95% CI)	2019 IR (95% CI) N = 463	RR (95% CI)
All events (community and hospital onset), rate per 100,000 population	8.08 (7.57–8.59)		8.54 (7.8–9.28)		7.63 (6.93–8.32)	
Males	9.34 (8.56–10.12)	1.36 (1.19–1.55)	9.85 (8.71–10.99)	1.35 (1.13–1.62)	8.84 (7.77–9.91)	1.37 (1.13–1.65)
Females	6.88 (6.22–7.53)	Reference	7.29 (6.33–8.25)	Reference	6.47 (5.58–7.37)	Reference
Community-onset events, rate per 100,000 population	2.64 (CI 2.35–2.93)		2.61 (2.2–3.02)		2.67 (2.26–3.08)	
Males	2.74 (2.32–3.17)	1.08 (0.86–1.36)	2.89 (2.28–3.51)	1.24 (0.89–1.73)	2.6 (2.02–3.18)	0.95 (0.69–1.31)
Females	2.54 (2.14–2.93)	Reference	2.33 (1.79–2.87)	Reference	2.74 (2.16–3.32)	Reference
Hospital-onset events, rate per 10,000 patient-days	0.62 (0.57–0.66)		0.67 (0.6–0.74)		0.57 (0.5–0.63)	
ICU wards	2.78 (2.26–3.29)	13.84 (10.08–19.16)	2.82 (2.08–3.56)	13.01 (8.35–20.55)	2.73 (2.02–3.45)	14.83 (9.31–24.09)
Medical wards	1.17 (1.06–1.28)	5.85 (4.48 -7.73)	1.27 (1.11–1.43)	5.85 (4.08–8.65)	1.08 (0.93–1.22)	5.84 (3.95–8.93)
Surgical wards	0.2 (0.15–0.25)	Reference	0.22 (0.14–0.29)	Reference	0.18 (0.12–0.25)	Reference

ICU—intensive care unit; CI—confidence interval; IR—incidence rate; RR—relative risk.

**Table 2:** Characteristics of *Acinetobacter baumannii* bloodstream infections, by place of onset.

	All Events N = 971	Hospital Onset (HO) N = 654	Community Onset (CO) N = 317	*p*-Value (HO vs. CO)
Patient characteristics				
Age, median (IQR) years	72 (62–83)	71 (61–82)	75 (64–84)	0.01
Male sex, no. (%)	548 (56.4)	387 (59.2)	161 (50.8)	0.02
Hospital Size *, no. (%)				
<300	NA	72 (11)	NA	
300–700		148 (22.6)		
>700		434 (66.4)		
Ward type, no. (%)				
ICU	NA	112 (17.1)	NA	
Medical		458 (70)		
Surgical		63 (9.6)		
Other		21 (3.2)		
Day of onset, median (IQR) days	NA	16 (9–30)	NA	
Season, no. (%)				
Winter	277 (28.5)	199 (30.4)	78 (24.6)	0.07
Spring	225 (23.2)	158 (24.2)	67 (21.1)	
Summer	227 (23.4)	141 (21.6)	86 (27.1)	
Autumn	242 (24.9)	156 (23.9)	86 (27.1)	
Polymicrobial event, no. (%)	196 (20.2)	132 (20.2)	64 (20.2)	1.0
Antibiotic susceptibility, no./total no. (%)				
AG	245/960 (25.5)	122/651 (18.7)	123/309 (39.8)	<0.001
Carb	225/961 (23.4)	97/652 (14.9)	128/309 (41.4)	<0.001
xCS	143/959 (14.9)	58/652 (8.9)	85/307 (27.7)	<0.001
FQ	180/958 (18.8)	71/652 (10.9)	109/306 (35.6)	<0.001
Sul	260/898 (29)	132/612 (21.6)	128/286 (44.8)	<0.001
BLBLI	170/955 (17.8)	68/650 (10.5)	102/305 (33.4)	<0.001
Fol	275/894 (30.8)	151/617 (24.5)	124/277 (44.8)	<0.001
MDR	779/961 (81.1)	577/652 (88.5)	202/309 (65.4)	<0.001
XDR	744/961 (77.4)	559/652 (85.7)	185/309 (59.9)	<0.001
PDR	521/961 (54.2)	404/652 (62)	117/309 (37.9)	<0.001
Outcome, no./total no. (%)				
14-day mortality	461/900 (51.2)	335/605 (55.4)	126/295 (42.7)	<0.001
30-day mortality	534/900 (59.3)	391/605 (64.6)	143/295 (48.5)	<0.001
1-year mortality	509/625 (81.4)	353/425 (83.1)	156/200 (78)	0.15

* Number of acute-care hospital beds. IQR—interquartile rage; NA—not applicable; ICU—intensive care unit; AG—aminoglycosides (amikacin, gentamicin, tobramycin); Carb—carbapenems (imipenem, meropenem); xCS—extended-spectrum cephalosporins (cefotaxime, ceftriaxone, ceftazidime, cefepime); FQ—fluoroquinolones (ciprofloxacin, ofloxacin, levofloxacin); Sul—ampicillin-sulbactam; BLBLI—antipseudomonal penicillins + β-lactamase inhibitors (piperacillin-tazobactam, ticarcillin-clavulanate); Fol—folate pathway inhibitors (trimethoprim-sulphamethoxazole); MDR—multidrug-resistant; XDR—extensively drug-resistant; PDR—pandrug-resistant.

**Table 3:** Characteristics of *Acinetobacter baumannii* bloodstream infections, by carbapenem resistance.

	CRAB N = 736	CSAB N = 225	*p*-Value
Patient characteristics			
Age, median (IQR) years	73 (64–83)	70 (57–82)	0.01
Male sex, no. (%)	428 (58.2)	116 (51.6)	0.09
Hospital onset, no. (%)	555 (75.4)	97 (43.1)	<0.001
Hospital Size *^, no./total no. (%)			
<300	58/555 (10.5)	14/97 (14.4)	0.01
300–700	116/555 (20.9)	31/97 (32)	
>700	381/555 (68.6)	52/97 (53.6)	
Ward type ^, no. /total no. (%)			
ICU	96/555 (17.3)	16/97 (16.5)	0.002
Medical	399/555 (71.9)	57/97 (58.8)	
Surgical	45/555 (8.1)	18/97 (18.6)	
Other	15/555 (2.7)	6/97 (6.2)	
Day of onset, median (IQR) ^ days	17 (10–31)	12 (7–22)	<0.001
Season, no. (%)			
Winter	239 (32.5)	36 (16)	<0.001
Spring	181 (24.6)	40 (17.8)	
Summer	151 (20.5)	74 (32.9)	
Autumn	165 (22.4)	75 (33.3)	
Polymicrobial event, no. (%)	146 (19.8)	49 (21.8)	0.57
Antibiotic susceptibility, no./total no. (%)			
AG	48/735 (6.5)	197/225 (87.6)	<0.001
xCS	3/734 (0.4)	140/225 (62.2)	<0.001
FQ	3/735 (0.4)	177/223 (79.4)	<0.001
Sul	73/699 (10.4)	187/199 (94)	<0.001
BLBLI	0/733 (0)	170/222 (76.6)	<0.001
Fol	129/706 (18.3)	146/188 (77.7)	<0.001
MDR	735 (99.9)	44 (19.6)	<0.001
XDR	730 (99.2)	14 (6.2)	<0.001
PDR	521 (70.8)	0 (0)	<0.001
Outcome, no./total no. (%)			
14-day mortality	417/685 (60.9)	43/207 (20.8)	<0.001
30-day mortality	474/685 (69.2)	59/207 (28.5)	<0.001
1-year mortality	434/490 (88.6)	72/130 (55.4)	<0.001

* Number of acute-care hospital beds. ^ Hospital-onset events only. CRAB—carbapenem-resistant *A. baumannii*; CSAB—carbapenem-susceptible *A. baumannii*; IQR—interquartile rage; ICU—intensive care unit; AG—aminoglycosides (amikacin, gentamicin, tobramycin); xCS—extended-spectrum cephalosporins (cefotaxime, ceftriaxone, ceftazidime, cefepime); FQ—fluoroquinolones (ciprofloxacin, ofloxacin, levofloxacin); Sul—ampicillin-sulbactam; BLBLI—antipseudomonal penicillins + β-lactamase inhibitors (piperacillin-tazobactam, ticarcillin-clavulanate); Fol—folate pathway inhibitors (trimethoprim-sulphamethoxazole); MDR—multidrug-resistant; XDR—extensively drug-resistant; PDR—pandrug-resistant.

## Data Availability

The data presented in this study are not publicly available due to privacy restrictions for healthcare data. Aggregate data will be available upon justified request.

## Supplementary Materials

The following supporting information can be downloaded at: https://www.mdpi.com/article/10.3390/microorganisms11092178/s1, Table S1: Predictors of 30-day mortality.
