# Supplementary material for: Acinetobacter baumannii Bloodstream Infections: A Nationwide Study in Israel
Source: Microorganisms. 2023 Aug 29;11(9):2178. doi: 10.3390/microorganisms11092178 (PMC10534809; doi:10.3390/microorganisms11092178)
Supplement: Supplementary file 1 [file microorganisms-11-02178-s001.zip › microorganisms-2530552-supplementary.pdf]

microorganisms

MDPI

Supplementary Materials:

Table S1. Predictors of 30-day mortality.

	**Alive N=366**	**Deceased N=534**	***p*-Value**
Patient characteristics			
Age, median (IQR) years	68.5 (57-78.25)	76 (66-85)	<0.001
Male sex, no. (%)	202 (55.2%)	310 (58.1%)	0.41
Hospital onset, no. (%)	214 (58.5%)	391 (73.2%)	<0.001
Hospital Size*^, no./total no, (%)			
<300	21/214 (9.8%)	44/391 (11.3%)	
300-700	49/214 (22.9%)	68/391 (17.4%)	0.25
>700	144/214 (67.3%)	279/391 (71.4%)	
Ward type^, no./total no, (%)			
ICU	48/214 (22.4%)	56/391 (14.3%)	
Medical	131/214 (61.2%)	297/391 (76%)	0.002
Surgical	28/214 (13.1%)	31/391 (7.9%)	
Other	7/214 (3.3%)	7/391 (1.8%)	
Day of onset, median (IQR)^ days	17 (10-32.25)	16 (9-28)	0.19
Polymicrobial event, no. (%)	74 (20.2%)	106 (19.9%)	0.93
Antibiotic susceptibility, no./total no. (%)			
AG	156/359 (42.6%)	74/532 (13.9%)	<0.001
Carb	148/359 (40.4%)	59/533 (11%)	<0.001
xCS	94/359 (25.7%)	38/531 (7.1%)	<0.001
FQ	128/358 (35%)	36/531 (6.7%)	<0.001
Sul	150/331 (41%)	94/505 (17.6%)	<0.001
BLBLI	112/357 (30.6%)	43/530 (8.1%)	<0.001
Fol	137/318 (37.4%)	119/513 (22.3%)	<0.001
MDR	232/359 (63.4%)	493/533 (92.3%)	<0.001
XDR	214/359 (58.5%)	478/533 (89.5%)	<0.001
PDR	139/359 (38%)	339/533 (63.5%)	<0.001

IQR- interquartile rage; ICU- intensive care unit; AG—aminoglycosides (amikacin, gentamicin, tobramycin); Carb—carbapenems (imipenem, meropenem); xCS—extended-spectrum cephalosporins (cefotaxime, ceftriaxone, ceftazidime, cefepime); FQ—fluoroquinolones (ciprofloxacin, ofloxacin, levofloxacin); Sul—ampicillin-sulbactam; BLBLI—antipseudomonal penicillins + β-lactamase inhibitors (piperacillin-tazobactam, ticarcillin-clavulanate); Fol—folate pathway inhibitors (trimethoprim-sulphamethoxazole); MDR—multidrug-resistant; XDR—extensively drug-resistant; PDR—pandrug-resistant.

* Number of acute-care hospital beds

^ Hospital onset events only
